# Factors affecting prognosis in patients treated with bevacizumab plus paclitaxel as first-line chemotherapy for HER2-negative metastatic breast cancer: an international pooled analysis of individual patient data from four prospective observational studies

**DOI:** 10.1007/s12282-022-01399-1

**Published:** 2022-09-03

**Authors:** Yutaka Yamamoto, Hiroyasu Yamashiro, Andreas Schneeweiss, Volkmar Müller, Oleg Gluz, Peter Klare, Bahriye Aktas, Dank Magdolna, László Büdi, Béla Pikó, László Mangel, Masakazu Toi, Satoshi Morita, Shinji Ohno

**Affiliations:** 1 Department of Breast and Endocrine Surgery, Kumamoto University Hospital, 1-1-1, Honjo, Chuo-ku, Kumamoto, 860-8556 Japan; 2 Department of Breast and Endocrine Surgery, Tenri Hospital, Tenri, Japan; 3 Heidelberg University Hospital and German Cancer Research Center, Heidelberg, Germany; 4 Department of Gynecology, Hamburg-Eppendorf University Medical Center, Hamburg, Germany; 5 Breast Center Niederrhein, Evangelical Hospital Johanniter Bethesda, Moenchengladbach, Germany; 6 Clinic for Cancer Medicine for Women/Breast Center, Berlin, Germany; 7 Department of Gynecology and Obstetrics, University of Essen, Essen, Germany; 8 Department of Gynecology, University of Leipzig, Leipzig, Germany; 9 1st Department of Internal Medicine Division of Oncology, Semmelweis University, Budapest, Hungary; 10 Clinical Oncology and Radiotherapy Center, B-A-Z County Hospital, Miskolc, Hungary; 11 Bekes County Pandy Kalman Hospital, Gyula, Hungary; 12 Medical School, University of Pecs, Pecs, Hungary; 13 Breast Surgery, Kyoto University Graduate School of Medicine, Kyoto, Japan; 14 Department of Biomedical Statistics and Bioinformatics, Kyoto University Graduate School of Medicine, Kyoto, Japan; 15 Center of Breast Oncology, The Cancer Institute Hospital of JFCR, Tokyo, Japan

**Keywords:** Bevacizumab, Paclitaxel, Metastatic breast cancer, Pooled analysis, Real-world evidence

## Abstract

**Background:**

Bevacizumab (BV) plus paclitaxel (PTX) is a treatment option in patients with HER2-negative metastatic breast cancer (mBC). We conducted an international pooled analysis with individual patient data to evaluate the effectiveness of BV + PTX as a first-line treatment for HER2-negative mBC patients under routine practice.

**Methods:**

A total of 2,474 mBC patients treated with BV + PTX from four prospective observational studies were analyzed. The primary endpoint was overall survival (OS). The other endpoints including identifying independent prognostic factors and validation of the modified Prognostic Factor Index (PFI) developed in the ATHENA trial.

**Results:**

Median follow-up time was 10.9 months (M). Median OS were 21.4 M (95% confidential interval 19.8–22.7 M). The seven independent prognostic factors (tumor subtype, age, ECOG performance status (PS), disease-free interval (DFI), liver metastases, number of metastatic organs, and prior anthracycline and/or taxane treatment) for OS found in this analysis included the five risk factors (RFs [DFI < 24 months, ECOG PS 2, liver metastases and/or > 3 metastasis organ sites, TNBC, prior anthracycline and/or taxane therapy]). High- (> 3 RFs [median OS 12.6 M]) and intermediate-risk groups (2 RFs [median OS 18.0 M]) had a significantly worse prognosis than the low-risk group (< 1 RF [median OS 27.4 M]), (*p* < 0.0001).

**Conclusions:**

This international pooled analysis showed the effectiveness of first-line BV + PTX for HER2-negative mBC patients identifying seven independent prognostic factors as real-world evidence. The usefulness of the modified PFI developed in the ATHENA trial in predicting OS among patients receiving BV + PTX was also verified.

**Supplementary Information:**

The online version contains supplementary material available at 10.1007/s12282-022-01399-1.

## Introduction

The prognosis of metastatic breast cancer is improving due to advances in treatment strategy with emerging novel modalities such as molecular targeted therapy [1]. However, metastatic breast cancer is still an incurable disease. In addition, the prognosis for metastatic breast cancer has been shown to vary by molecular subtype [2]. Even today, the goal of treatment for metastatic breast cancer is to improve and maintain quality of life through improving cancer-related symptom, as well as to prolong survival.

Bevacizumab is a recombinant humanized monoclonal antibody against human vascular endothelial growth factor (VEGF), a multifunctional and most important cytokine among angiogenic factors. VEGF regulates cell division and vascular endothelial cell survival and is also involved in increasing vascular permeability [3]. Through these actions, VEGF promoted tumor growth, progression and metastasis. Its overexpression has been observed in many types of tumors [4]. Recently, it has also been reported that VEGF acts suppressively on the immune system via various pathways [5].

The results of a meta-analysis using seven randomized controlled trials [6] showed that the addition of bevacizumab to chemotherapy as first-line chemotherapy for metastatic breast cancer significantly prolonged PFS [hazard ratio (HR): 0.72, 95% confidential interval (CI) 0.67–0.77, *P* < 0.00001]., increased objective response rates (ORR) (HR: 1.47, 95% CI 1.26–1.71, *P* < 0.00001), and did not prolong overall survival (OS) compared to chemotherapy alone. Moreover, the addition of bevacizumab significantly increased the incidence of treatment discontinuation due to toxicity (HR: 1.43, 95% CI 1.06–1.93, *P* = 0.02) and Grade 3 or higher adverse events (HR: 1.43; 95% CI 1.25–1.64, *P* < 0.00001).

On the other hand, an observational study using a large database of metastatic breast cancer patients in France reported that bevacizumab in combination with paclitaxel as first-line chemotherapy significantly prolonged OS compared to paclitaxel alone [7]. In another meta-analysis using randomized controlled trials, the addition of bevacizumab to chemotherapy significantly reduced the 1-year mortality rate of metastatic breast cancer patients and improved the OS in patients treated with neoadjuvant or adjuvant taxane [8].

Recently, large-scale observational studies are becoming increasingly important as complementary tools for randomized control trials, several non-interventional studies have evaluated the effectiveness and safety of bevacizumab plus paclitaxel for HER2-negative advanced breast cancer patients in the real world [9]. We conducted this pooled analysis with individual patient data from four prospective observational studies, ML21165 [10], ML21647 [11], ML22452 [12] and B-SHARE [13], to more accurately estimate the effectiveness of bevacizumab plus paclitaxel and to evaluate effectiveness in selected subgroups such as triple-negative subtype. We also tried to identify prognostic factors in terms of survival in this pooled analysis. In addition, we validated the Prognostic Factor Index developed in the ATHENA trial [14] with this pooled analysis data.

## Material and methods

### Data collection

This study is a collaborative pooled analysis using independent patient data from four prospective non-interventional studies to assess the effectiveness of bevacizumab in combination with paclitaxel as first-line chemotherapy for metastatic breast cancer. These four observational studies included ML21165 [10], ML21647 [11], ML22452 [12], and B-SHARE [13], (Supplementary Table 1). The main results of these four studies have already been published individually [10–13]. This pooled analysis was conducted by the Japan Breast Cancer Research Group (JBCRG) as a sub-study of the B-SHARE (UMIN ID: UMIN000047290). The transfer of the data of German (ML21165 and ML22452) and Hungarian (ML21647) trials to the JBCRG was carried out as follows. The F. Hoffmann-La Roche, Ltd received approval from the principal investigators of the German and Hungarian trials to transfer the data for each trial to the JBCRG. The JBCRG requested the F. Hoffmann-La Roche, Ltd to transfer the data of the three trials for this pooled analysis. The data of German and Hungarian studies were transferred to JBCRG after the data sharing agreement was signed between the JBCRG and F. Hoffmann-La Roche, Ltd. In the process of consenting to each study, patients agreed to use data for further medical research from other countries.

### Study population

The inclusion criteria of this pooled analysis were patients with HER2-negative metastatic breast cancer who had not received prior chemotherapy for metastatic breast cancer. The exclusion criteria of this analysis were patient with locally advanced breast cancer and local recurrence without distant metastasis, and patients who have never received bevacizumab plus paclitaxel for metastatic breast cancer. The following data were collected from the four studies for this analysis: age, performance status, estrogen receptor (ER) status, progesterone receptor (PgR) status, HER2 status, history of surgery for primary disease, history of adjuvant systemic treatment, disease-free interval, metastatic site, number of metastatic organs, date of beginning and end of bevacizumab plus paclitaxel, date of progression and date of death. ER-positive and/or PgR was defined as hormone receptor status. ER-positive/PgR-positive was determined differently for each study. In the B-SHARE, ER-positive/PgR-positive was defined by occupancy of stained positive nuclear in tumor cells of 1% or more by immunohistochemical staining. In the other three studies (ML21165, ML22452 and ML21647), ER-positive/PgR-positive was determined by pathologist in each institute according to local guidelines in each country. Visceral metastasis was defined as organ metastasis excluding skin, soft tissue, lymph node and bone.

Bevacizumab was given in combination with paclitaxel according to the physician’s standard practice. In general, the treatment schedule of paclitaxel and bevacizumab was given according to the licensed label in Germany (ML21165 and ML22452) and Hungary (ML21647) as follows: bevacizumab 10 mg/kg every 2 weeks or 15 mg/kg every 3 weeks combined with paclitaxel administered weekly or every 3 weeks. On the other hand, in Japan (B-SHARE), the treatment regimen recommended the following standard treatment regimen: bevacizumab 10 mg/kg given every 2 weeks in combination with paclitaxel 90 mg/m^2^ given every week for 3 weeks, followed by a 1-week rest. Both drugs were continued until disease progression, unacceptable toxicity, or withdrawal of consent.

The primary endpoint is OS. OS was defined as from the beginning of bevacizumab plus paclitaxel to the date of death from any cause. The secondary endpoints are PFS, ORR and validation of the Prognostic Factor Index developed in the ATHENA trial [14]. Response and disease progression was assessed according to the investigator assessment. PFS was defined as from the beginning of bevacizumab plus paclitaxel to the date of progression or death from any cases. Time to treatment failure (TTF) was defined as from the beginning of bevacizumab plus paclitaxel to discontinuation of this treatment for any reason included disease progression, unacceptable adverse events, any cause of death and withdrawal consent. OS, PFS, TTF and ORR were evaluated by all eligible patients and by hormone receptor status.

We also tried to validate the Prognostic Factor Index composed of risk factors identified in the ATHENA study [14]. The risk factors are as follows: disease-free interval < 24 months; liver metastases or 3 involved organ sites; prior anthracycline and/or taxane therapy; triple-negative breast cancer; and performance status 2 or prior analgesic/corticosteroid treatment. This analysis did not include the presence or absence of analgesic and corticosteroid use. Because the data set in this pooled analysis lacked data on the use of analgesic and corticosteroids. The modified prognostic factor index in this study is as follows: patient with 0 or 1 risk factor was defined as low risk, patients with 2 risk factors were defined as intermediate risk, and patients with 3 or more risk factors as high risk.

### Statistical analysis

The cumulative survival curve for OS, median OS, and survival rates in each year were estimated using the Kaplan–Meier method, and Greenwood’s formula was used to construct 95% CIs. The log-rank test was used to compare the survival curves of the two study arms. Subgroup analysis was performed by Cox regression analysis to identify important prognostic factors. The factor used for multivariate analysis is *p* < 0.05 in univariate analysis. The same analyses were performed for PFS as those for OS. ORR was calculated as the proportion of patients achieving complete or partial response as the best overall response in patients whose response rate was evaluated in the individual studies. CIs were calculated using the Clopper–Pearson method. There was no adjustment for multiple testing.

Statistical analysis was performed using SAS^®^ version 9.4 (SAS, Cary NC, USA). *P* values of < 0.05 were considered statistically significant.

## Results

### Study population and characteristics of the patients

From the four prospective observational studies [10–13], 2902 patients were included in the dataset for this pooled analysis. Among them, 2474 HER2-negative metastatic breast cancer patients were used in this pooled analysis, excluding patients not receiving bevacizumab plus paclitaxel as first-line chemotherapy (*n* = 279), locally advanced or local recurrent breast cancer without distant metastasis (n = 184), and patients with other reason for ineligibility (*n* = 13) (Supplementary Fig. 1).

Baseline patients’ characteristics are summarized in Table 1 and Supplementary Table 2. Median age was 59 years. ECOG performance status (PS) 2 or above was 201 (8.3%). Hormone receptor-positive, triple-negative and unknown subtype in HER2-negative breast cancer accounted for 1806 (73.0%), 544 (22.0%), and 124 (5.0%), respectively. Recurrence within 24 months after treatment for primary breast cancer was included in 412 (16.7%) cases. Cases with visceral metastasis, with ≥ 3 metastatic organs, liver metastases, lung metastases and bone metastases were 1843 (74.5%), 463 (18.7%), 1029 (41.6%), 939 (38.0%), and 1353 (54.7%), respectively. About half of the cases (1402, 56.7%) were treated with adjuvant chemotherapy. Of these, most cases (1239, 88.4%) were treated with anthracyclines and/or taxanes.

**Table 1:** Patients’ characteristics

	All patients		HR-positive		Triple-negative		Unclassified	
	*N*	(%)	*N*	(%)	*n*	(%)	*N*	(%)
No. of patients	2474	100%	1806	100%	544	100%	124	100%
Median age (range), years	59.0	(24–87)	59.4	(24–86)	56.3	(26–83)	64.5	(28–87)
ECOG PS								
0	1246	50.4%	902	49.9%	303	55.7%	41	33.1%
1	932	37.7%	700	38.8%	170	31.3%	62	50.0%
> 2	201	8.1%	141	7.9%	50	9.2%	10	8.1%
Missing	95	3.8%	63	3.5%	21	3.9%	11	8.9%
ER status of primary tumor								
Negative	667	27.0%	108	6.0%	537	98.7%	22	17.7%
Positive	1689	68.3%	1682	93.1%	7	1.3%	0	0.0%
Missing/Unknown	118	4.7%	16	0.9%	0	0.0%	102	82.3%
PgR status of primary tumor								
Negative	878	35.5%	317	17.6%	541	99.4%	20	16.1%
Positive	1463	59.1%	1460	80.8%	3	0.6%	0	0.0%
Unknown	133	5.4%	29	1.6%	0	0.9%	104	83.9%
HER2 status of primary tumor								
Negative	1994	80.6%	1432	79.3%	542	99.6%	20	16.1%
Positive	161	6.5%	161	8.9%	0	0.0%	0	0.0%
Missing/Unknown	319	12.9%	213	11.8%	2	0.4%	2	1.6%
Disease-free interval, months								
< 24 (recurrence)	412	16.7%	214	11.9%	189	34.7%	9	7.3%
> 24 (recurrence) or 0 (de novo)	2026	81.9%	1562	86.5%	350	64.3%	114	91.9%
Missing	36	1.4%	30	1.6%	5	1.0%	1	0.8%
Metastatic site								
Non-visceral	631	25.5%	450	24.9%	145	26.7%	36	29.0%
Visceral	1843	74.5%	1356	75.1%	399	73.3%	88	71.0%
No. of metastatic organs								
< 3	2011	81.3%	1448	80.2%	463	85.1%	100	80.6%
> 3	463	18.7%	358	19.8%	81	14.9%	24	19.4%
Metastatic sites^a^								
Liver	1029	41.6%	817	45.2%	172	31.6	40	32.3
Lung	939	38.0%	611	33.8%	275	50.6%	53	42.7%
Central nervus system	52	2.3%	28	1.6%	22	4.0%	6	4.8%
Bone	1353	54.7%	1083	60.0%	204	37.5%	66	53.2%
Bone only	312	12.6%	252	14.0%	47	8.6%	13	10.5%
Other	957	38.7%	660	36.5%	236	43.3%	61	49.2%
History of surgery for primary disease								
No	1179	47.7%	844	46.7%	279	51.3%	56	45.2%
Yes	1168	47.2%	870	48.2%	230	42.3%	68	54.8%
Missing	127	5.1%	92	5.1%	35	6.4%	0	0.0%
History of adjuvant therapy								
Chemotherapy	1402	56.7%	976	54.0%	373	68.6%	53	42.7%
Anthracycline (A)^b^	1193	85.1%	823	84.3%	339	90.9%	31	58.5%
Taxane (T)^b^	686	48.9%	454	46.5%	222	59.5%	10	18.9%
A and/or T^b^	1239	88.4%	967	99.1%	356	95.4%	31	58.5%
Endocrine therapy	1280	51.7%	1137	63.0%	85	15.8%	57	46.0%
Radiotherapy	1419	57.4%	1073	59.4%	270	49.6%	76	61.3%

*ECOG PS* Eastern Cooperative Oncology Group performance status, *ER* estrogen receptor, *HR* hormone receptor, *PgR* progesterone receptor

^a^Multiple items could be selected

^b^Number (%) of patients treated with adjuvant chemotherapy

Median follow-up time was 10.9 month (interquartile range [IQR] 5.5–20.0 months). In addition, median treatment period with bevacizumab plus paclitaxel was 5.6 months (IQR 3.2–9.8). Treatment was discontinued in 2176(88.8%) cases during the observation period. At the end of the observation period, 298(12.0%) patients continued treatment. Treatment was discontinued due to disease progression in 1,220(56.1%), adverse events in 277(12.7%), and other reasons in 551(25.3%) patients (Supplementary Table 1).

### Overall survival

**Fig. 1:**
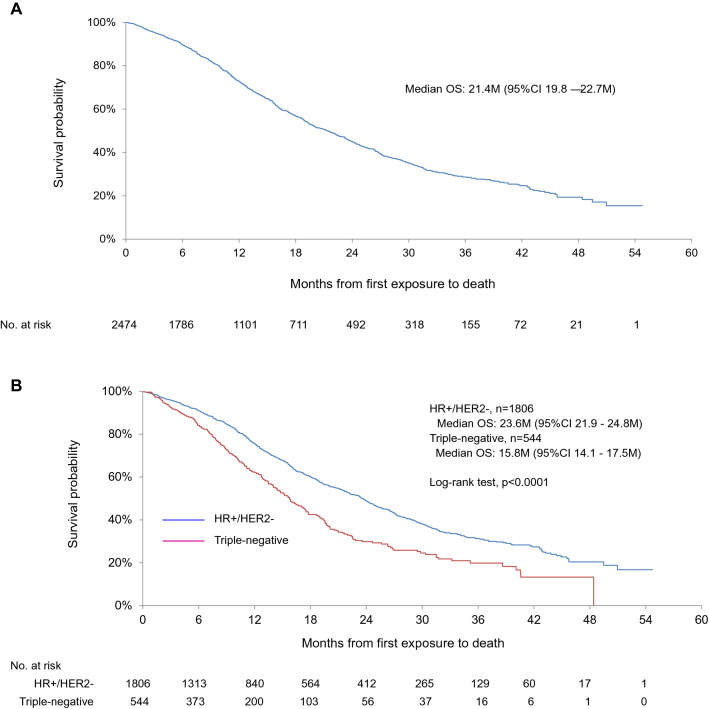
Overall survival in HER2-negative MBC patients treated with bevacizumab in combination with paclitaxel as first-line chemotherapy: **a** whole population; **b** hormone receptor-positive breast cancer vs. triple-negative breast cancer

The number of OS events during the observational period was 1064 (43.0%) cases. Median OS in the whole population, hormone receptor-positive, and triple-negative subtype was 21.4 months (95% CI, 19.8 – 22.7), 23.6 months (95% CI 21.9–24.8), and 15.8 months (95% CI 14.1–17.5), respectively (Fig. 1a and b). 1-year OS in the whole population, hormone receptor-positive, and triple-negative subtype was 72.7%, 75.5%, and 62.4%, respectively. OS in the hormone receptor-positive cohort had significantly longer than that in a triple-negative cohort (log-rank test, *p* < 0.0001, Fig. 1b).

Multivariate analysis identified the following baseline characteristics independently associated with poor OS: triple-negative subtype, 70 or more years old, ECOG PS 2 or more, disease-free survival within 24 months, liver metastasis, no lung metastasis, 3 or more metastatic organs, and history of neoadjuvant or adjuvant taxane and/or anthracycline (Table 2).

**Table 2:** Results of univariate and multivariate analyses for overall survival

			Median survival time (M)		Univariate analysis			Multivariate analysis		
Variables	Categories	No.	Estimate	95% CI	HR	95% CI	*P*	HR	95% CI	*P*
Subtype	HR-positive/HER2-negative Triple-negative	1806 544	23.6 15.8	21.9–24.8 14.1–17.5	Ref 1.61	1.40–1.85	< 0.0001	Ref 1.58	1.34–1.86	< 0.0001
Age	< 70 years old > 70 years old	2035 439	22.3 16.5	20.7–23.79 14.8–19.8	Ref 1.42	1.22–1.66	< 0.0001	Ref 1.55	1.59–1.88	< 0.0001
ECOG PS	0 > 2	2178 201	22.3 12.7	21.0–23.8 10.7–16.0	Ref 1.87	1.55–2.26	< 0.0001	Ref 1.91	1.57–2.34	< 0.0001
Disease-free interval	< 24 M > 24 M or 0 M (de novo)	251 2201	13.4 22.7	11.7–15.8 21.0–24.1	Ref 0.56	0.48–0.66	< 0.0001	Ref 0.68	0.58–0.80	< 0.0001
Visceral metastasis	No Yes	631 1843	23.8 20.6	19.9–26.4 19.2–22.2	Ref 1.20	1.04–1.39	0.0011	Ref 0.99	0.78–1.25	0.9210
Bone metastasis only	No Yes	2162 312	21.5 19.9	19.8–22.8 17.8–25.23	Ref 1.02	0.85–1.12	0.8714			
Liver metastasis	No Yes	1445 1029	25.5 17.2	22.5–26.5 15.9–19.0	Ref 1.60	1.42–1.80	< 0.0001	Ref 1.55	1.28–1.88	< 0.0001
Lung metastasis	No Yes	1535 939	19.9 23.3	18.6–21.8 21.0–26.5	Ref 0.84	0.74–0.95	0.0053	Ref 0.86	0.71–1.04	0.1172
No. of metastatic organs	< 3 > 3	2011 463	22.5 17.8	21.0–24.0 16.0–19.5	Ref 1.34	1.16–1.55	0.0001	Ref 1.36	1.14–1.64	0.0009
Neoadjuvant or adjuvant taxane or anthracycline	No Yes	1084 1239	26.5 17.7	24.2–28.2 16.4–18.9	Ref 1.54	1.35–1.75	< 0.0001	Ref 1.41	1.23–1.63	< 0.0001

*CI* confidence interval, *ECOG PS* Eastern Cooperative Oncology Group performance status, *HR* hazard ratio, *HR-positive* hormone receptor-positive, *M* month, *No* number

### Secondary endpoints

**Fig. 2:**
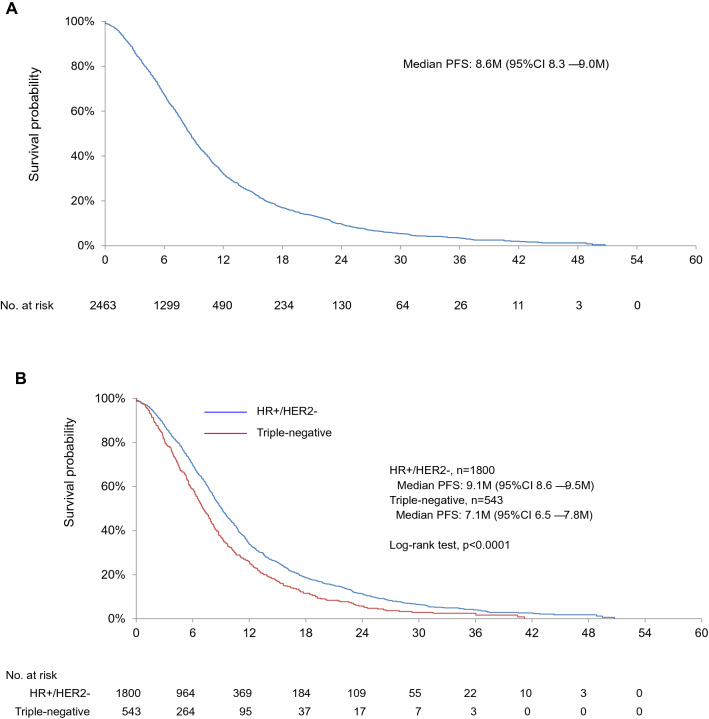
Progression-free survival in HER2-negative MBC patients treated with bevacizumab in combination with paclitaxel as first-line chemotherapy: **a** whole population; **b** hormone receptor-positive breast cancer vs. triple-negative breast cancer

In the analysis of PFS and TTF, 2,463 cases were used, excluding 11 cases who did not start BV + PTX treatment as 1st line chemotherapy (Supplementary Fig. 1). The number of PFS events during the observational period was 1709 (69.4%) cases. Median PFS in the whole population, in hormone receptor-positive cohort, and in the triple-negative cohort was 8.6 months (95% CI 8.3–9.0 months), 9.1 months (95% CI 8.6–9.5 months), 7.1 months (95% CI, 6.5–7.8 months), respectively (Fig. 2a and b). PFS in hormone receptor-positive cohort had significantly longer than that in a triple-negative cohort (log-rank test, *p* < 0.0001, Fig. 2b).

Multivariate analysis identified the following baseline characteristics independently associated with poor PFS (Table 3): triple-negative subtype, ECOG PS ≥ 2, disease-free survival within 24 months, liver metastasis, and history of neoadjuvant or adjuvant chemotherapy.

**Table 3:** Results of univariate and multivariate analyses for progression-free survival

			Median survival time (M)		Univariate analysis			Multivariate analysis		
Variables	Categories	No.	Estimate	95% CI	HR	95% CI	*P*	HR	95% CI	*P*
Subtype	HR-positive/HER2-negative Triple-negative	1800 543	9.6 7.6	9.2–10.4 6.7–8.2	Ref 1.36	1.19–1.55	< 0.0001	Ref 1.35	1.16–1.56	< 0.0001
Age	< 70 years old > 70 years old	2028 437	9.2 9.2	8.7–9.7 8.3–10.4	Ref 1.07	0.92–1.24	0.4129			
ECOG PS	0 > 2	2171 200	9.3 6.2	9.0–10.0 5.3–7.5	Ref 1.76	1.46–2.12	< 0.0001	Ref 1.77	1.45–2.17	< 0.0001
Disease-free interval	< 24 M > 24 M or 0 M (de novo)	251 2192	6.6 9.6	5.8–7.4 9.2–10.3	Ref 0.59	0.51–0.70	< 0.0001	Ref 0.69	0.59–0.80	< 0.0001
Visceral metastasis	No Yes	627 1838	9.2 9.1	8.3–10.7 8.7–9.7	Ref 1.09	0.96–1.25	0.1928			
Bone metastasis only	No Yes	2155 310	9.2 8.4	8.8–9.7 7.4–10.4	Ref 1.07	0.89–1.28	0.4378			
Liver metastasis	No Yes	1440 1025	10.1 8.4	9.2–11.0 8.0–8.9	Ref 1.40	1.25–1.56	< 0.0001	Ref 1.36	1.19–1.56	< 0.0001
Lung metastasis	No Yes	1529 936	8.8 10.1	8.3–9.3 9.0–11.4	Ref 0.82	0.73–0.93	0.0012	Ref 0.87	0.75–1.01	0.0587
No. of metastatic organs	< 3 > 3	2003 462	9.2 8.7	8.8–9.7 7.4–9.7	Ref 1.20	1.04–1.38	0.0120	Ref 1.14	0.96–1.36	0.1336
Neoadjuvant or adjuvant taxane or anthracycline	No Yes	1080 1234	10.8 8.3	9.7–11.6 7.8–8.7	Ref 1.43	1.27–1.60	< 0.0001	Ref 1.30	1.14–1.48	< 0.0001

*CI* confidence interval, *ECOG PS* Eastern Cooperative Oncology Group performance status, *HR* hazard ratio, *HR-positive* hormone receptor-positive, *M* month, *No* number

Median TTF in the whole population, hormone receptor-positive subtype, and triple-negative subtype were 6.1 months (95% CI 5.8–6.3), 6.2 months (95% CI 6.0–6.6), and 5.3 months (95% CI 4.9–5.6), respectively (Supplementary Fig. 2a and b). TTF in the hormone receptor-positive cohort had significantly longer than that in a triple-negative cohort (log-rank test, *p* < 0.0001, Supplementary Fig. 2b).

ORR was 54.3%, 55.1%, and 49.0% in the whole population, in hormone receptor-positive subtype, and in the triple-negative subtype, respectively. (Table 3). ORR in the hormone receptor-positive cohort had significantly higher than that in a triple-negative cohort (log-rank test, *p* < 0.0001, Table 4).

**Table 4:** Overall response rate in patients with evaluable lesions

	All		HR-positive/HER2-negative		Triple-negative		*P*
No. of patients	2474		1806		544		
No. of patients with evaluable lesions	1829	(100.0%)	1319	(100.0%)	412	(100.0%)	
Best response, *n* (%)							
CR	137	(7.5%)	86	(6.5%)	41	(10.0%)	*P* < 0.0001
PR	856	(46.8%)	641	(48.6%)	161	(39.1%)	
SD	512	(28.0%)	377	(28.6%)	112	(27.2%)	
PD	324	(17.7%)	215	(16.3%)	98	(23.8%)	
NE/missing	645		487		132		
Response rate, *n* (%)							
CR plus PR	993	(54.3%)	727	(55.1%)	202	(49.0%)	0.0316
95% CI	52.0–56.6%		52.4–57.8%		44.1–54.0		

*CR* complete response, *NE* not evaluable, *PD* progressive disease, *PR* partial response, *SD* stable disease

### Validation of the modified Prognostic Index developed in the ATHENA study

**Fig. 3:**
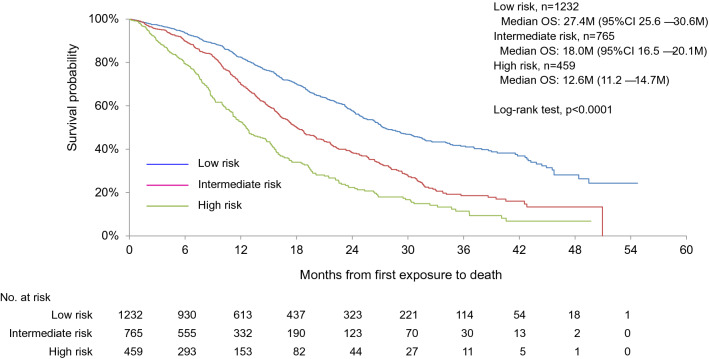
Overall survival according to risk group defined by the Prognostic Factor Index developed in the ATHENA study for HER2-negative MBC patients treated with bevacizumab in combination with paclitaxel as first-line chemotherapy

This validation of modified Prognostic Factor Index was conducted using 2456 (99.3%) cases excluding 17 cases without information of 5 risk factors from the total number of cases. Among them, patients with low risk (0 or 1 risk factor), intermediate risk (2 risk factors), and high risk (> 3 risk factors) were 1232 (50.2%), 765 (31.1%), and 459 (18.7%). Figure 3 shows that OS of patients with intermediate (median OS 18 months, 95% CI 16.5–20.1) and high risk (median OS 12.6 months, 95% CI 11.2–14.7 months) were significantly worse than that of patients with low risk (median OS 27.4 months, 95% CI 25.6–30.6 [low vs. intermediate risk: HR 1.76, 95% CI 1.53–2.03; low vs. high risk: HR 2.85, 95% CI 2.44–3.33]). Kaplan–Meier curves for OS in patients treated with bevacizumab plus chemotherapy is stratified for each risk group by the modified Prognostic Factor Index.

## Discussion

This international pooled analysis with four prospective observational studies was to examine the effectiveness of bevacizumab plus paclitaxel as first-line chemotherapy for HER2-positive breast cancer. As the real-world evidence of first-line bevacizumab plus paclitaxel for HER2-negative metastatic breast cancer, median OS and PFS was 21.4 months (95% CI 19.8–22.7) and 8.6 months (95% CI 8.3–9.0 M), respectively. 1-year OS and ORR were also 72.7% and 54.3%, respectively.

The ORR in this pooled analysis was similar to that in bevacizumab plus paclitaxel in previously reported randomized controlled trials (RCTs) [15–19]; Median ORR in these RCTs ranged from 36.9 to 54.0%. However, the OS and PFS in this pooled analysis seem to be shorter than these RCTs [15–17, 20] and large-scale observational studies [7, 21, 22]. The median OS in these RCTs has been reported in the range of 25.2 months and 30.2 months [15–20]. The median PFS in these RCTs has been also reported in the range of 9.2 months and 11.8 months [15–20]. Differences in OS and PFS between this pooled analysis and the RCTs may explain by differences in the patients’ background and in the medical care of participating patients in these studies. Poor PS has been reported to be an independent prognostic factor in metastatic breast cancer patients with [13, 14, 21] or without [22] bevacizumab use. Patients with ECOG PS 2 were not enrolled in these RCTs [15–17]. Prognosis of MBC patients may be affected by the better general condition in patients who can participate in RCTs than in clinical practice. Patients with ECOG PS 2 or more in this pooled analysis and the ATHENA study [9] was 8.1% and 5.5%, respectively. In addition, only MBC patients were analyzed in this pooled analysis, but local recurrent breast cancer was registered in addition to metastatic disease in the ATHENA study [9]. In general, MBC patients have a worse prognosis than patients with local recurrent BC [24, 25].

In addition, the duration of treatment in this pooled analysis (5.6 months) was shorter than that in the RCTs (6.9–8.0 months) [9, 18]. A reason to explain this difference is that the four studies used in this pooled analysis were conducted in routine practice and could be discontinued at the patient's wish or at the discretion of the attending physician, regardless of whether treatment remained effective or unacceptable adverse events.

Median OS (23.6 months) and PFS (9.1 months) in hormone receptor-positive BC were significantly longer than those in triple-negative BC (OS 15.8 months, PFS 7.1 months) in this pooled analysis. These prognostic differences in patients treated with bevacizumab plus paclitaxel are consistent with previously published data [13, 14, 26, 27]. Multivariate analysis for OS also identified several baseline characteristics as independent poor prognostic factors in this pooled analysis: TNBC, > 70 years old, ECOG PS > 1, disease-free interval < 24 months, liver metastasis, > 3 metastatic organs, and neoadjuvant or adjuvant taxane/anthracycline. These factors are almost consistent with independent poor prognostic factors identified in the ATHENA study [14]. Furthermore, these factors are not only prognostic factors for patients who received bevacizumab plus chemotherapy but also prognostic factors for patients treated with first-line chemotherapy without bevacizumab [23, 28]. On the other hand, a metaanalysis of three RCTs including E1100 [15], AVADO [29], RIBBON-1 [16] trials showed that adding bevacizumab to chemotherapy improved PFS in MBC patients with these poor prognostic factors compared to chemotherapy alone [8]. It was also shown that the addition of bevacizumab significantly improved 1-year OS and OS in patients previously treated with neoadjuvant or adjuvant taxane [8]. Thus, even in MBC patients with poor prognostic factors, the addition of bevacizumab to chemotherapy may be more effective than chemotherapy alone.

To further stratify the prognosis among HER2-negative advanced breast cancer patients, the Prognostic Factor Index was exploratorily developed in the ATHENA study [14]. We tried to validate the modified Prognostic Factor Index using almost the same number of cases as in the ATHENA study (*n* = 2203) in this pooled analysis (*n* = 2456). The modified Prognostic Factor Index could clearly stratify the OS of this pooled analysis population by risk group in the ATHENA study (Fig. 3). HR for OS in the low-risk group to that in intermediate (1.76, 95% CI 1.53–2.03) and high-risk group (2.85, 95% CI 2.44–3.33) were similar to the ATHENA study [14]. It was validated that OS in HER2-negative MBC patients treated with bevacizumab plus chemotherapy is stratified by each risk group by the Prognostic Factor Index developed in the ATHENA study [14]. Even bevacizumab plus paclitaxel has not achieved satisfactory outcomes for HER2-negative MBC patients with multiple risk factors. New treatment strategies are needed for MBC patients with multiple risk factors to further improve prognosis.

This pooled analysis included several limitations. First, this pooled analysis collected individual data from patients treated with bevacizumab plus paclitaxel for MBC in four prospective single-arm studies. Therefore, it was not possible to directly examine the efficacy of comparing bevacizumab plus paclitaxel and paclitaxel alone. Second, the category and duration of data collection in each observational study were different for each study because the primary endpoint in these four studies was different as follows; The ML21165 [10] and AVANTI (ML22452) [12] studies had no primary endpoint but prespecified endpoints such as safety and effectiveness (ORR, PFS and OS), and primary endpoint in the AVAREG (ML21647) [11] and the B-SHARE [13] studies were PFS and OS, respectively. Therefore, the median observation period at the time of this pooled analysis was relatively short. Third, the effectiveness of treatment (PFS and ORR) was assessed by attending physicians with inconsistent diagnostic methods for progression and follow-up intervals. Hormone receptor and HER2 status were also assessed at each institute. Central assessment or review was not done for the evaluation of effectiveness and those receptors status. Fourth, there was no data on subsequent treatment after bevacizumab plus paclitaxel in this pooled analysis, although OS, the primary endpoint of this pooled analysis, is a robust endpoint compared to PFS and ORR under routine oncology practice. However, it has not been proven that the prognosis after progression varies depending on the type of subsequent treatment after initial bevacizumab plus taxane [30, 31].

In conclusion, this international large-scale pooled analysis shows that the effectiveness of first-line bevacizumab plus paclitaxel for HER2-negative MBC patients in routine clinical practice and identified independent prognostic factors, tumor subtype, age, ECOG PS, disease-free interval, liver metastasis, number of metastatic organs, and prior taxane and/or anthracycline use, in terms of OS. In addition, the usefulness of the PI developed in the ATHENA trial in predicting the prognosis among patients receiving BV + PTX was verified in this pooled analysis. New treatment strategies are needed for MBC patients with multiple risk factors to further improve prognosis.

## Supplementary Information

Below is the link to the electronic supplementary material.

Supplementary file1 (PDF 89 KB)

Supplementary file2 (DOCX 43 KB)

## Data Availability

All data generated or analyzed in this study are available from the corresponding author.

## Abbreviations

BV: Bevacizumab
CI: Confidential interval
DFI: Disease-free interval
ER: Estrogen receptor
ECOG: Eastern Cooperative Oncology Group
HER2: Human epidermal growth factor receptor 2
HER2-: HER2-negative
HR: Hazard ratio
HR +: Hormone receptor-positive
mBC: Metastatic breast cancer
ORR: Objective response rate
OS: Overall survival
PFI: Prognostic factor index
PFS: Progression-free survival
PgR: Progesterone receptor
PS: Performance status
PTX: Paclitaxel
RCT: Randomized controlled study
RF: Risk factor
TNBC: Triple-negative breast cancer
