# Supplementary material for: Factors affecting prognosis in patients treated with bevacizumab plus paclitaxel as first-line chemotherapy for HER2-negative metastatic breast cancer: an international pooled analysis of individual patient data from four prospective observational studies
Source: Breast Cancer. 2022 Sep 3;30(1):88–100. doi: 10.1007/s12282-022-01399-1 (PMC9813142; doi:10.1007/s12282-022-01399-1)
Supplement: Supplementary file 1 — Supplementary file1 (PDF 89 KB) [file 12282_2022_1399_MOESM1_ESM.pdf]

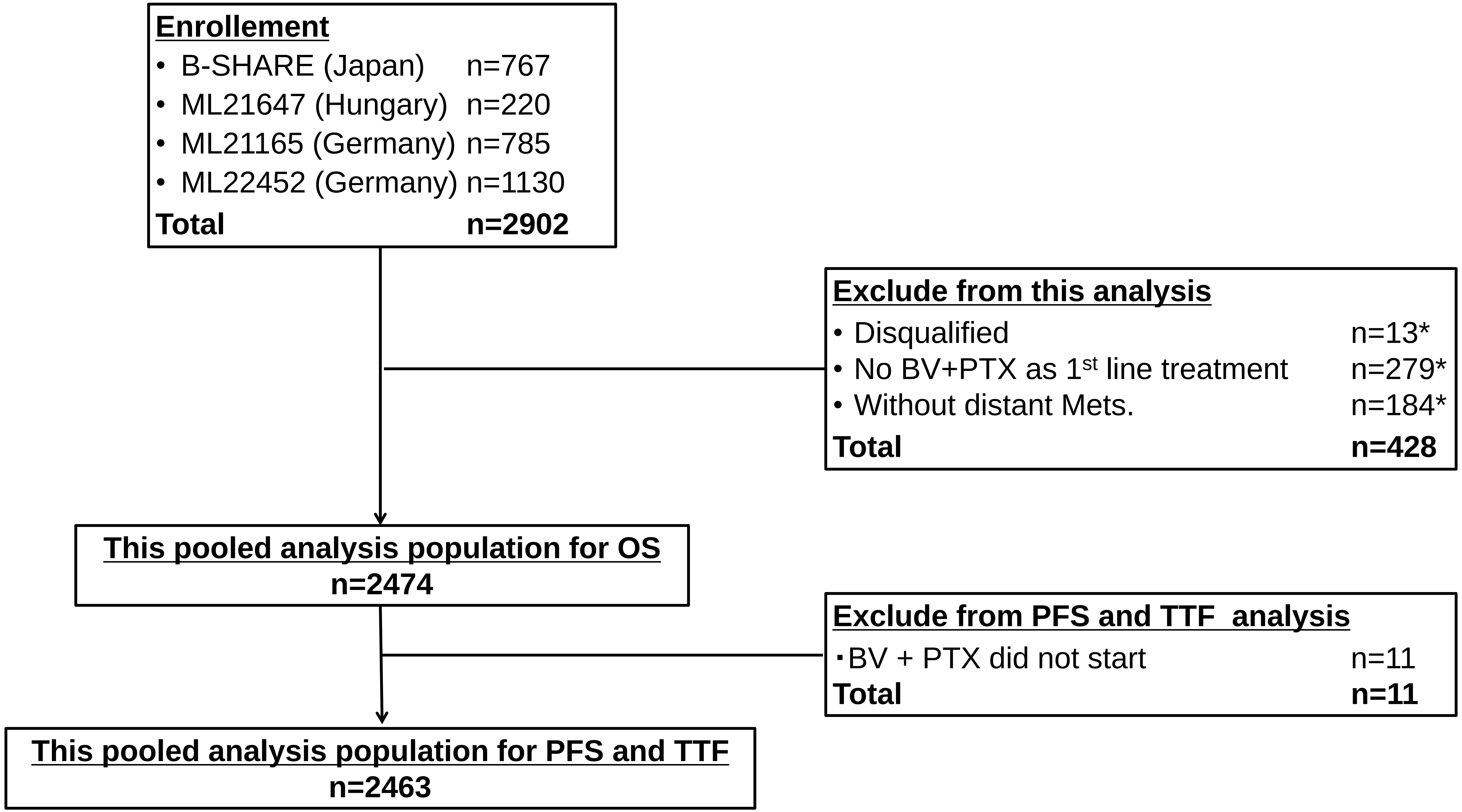

*Multiple items could be selected

Supplementary figure 1

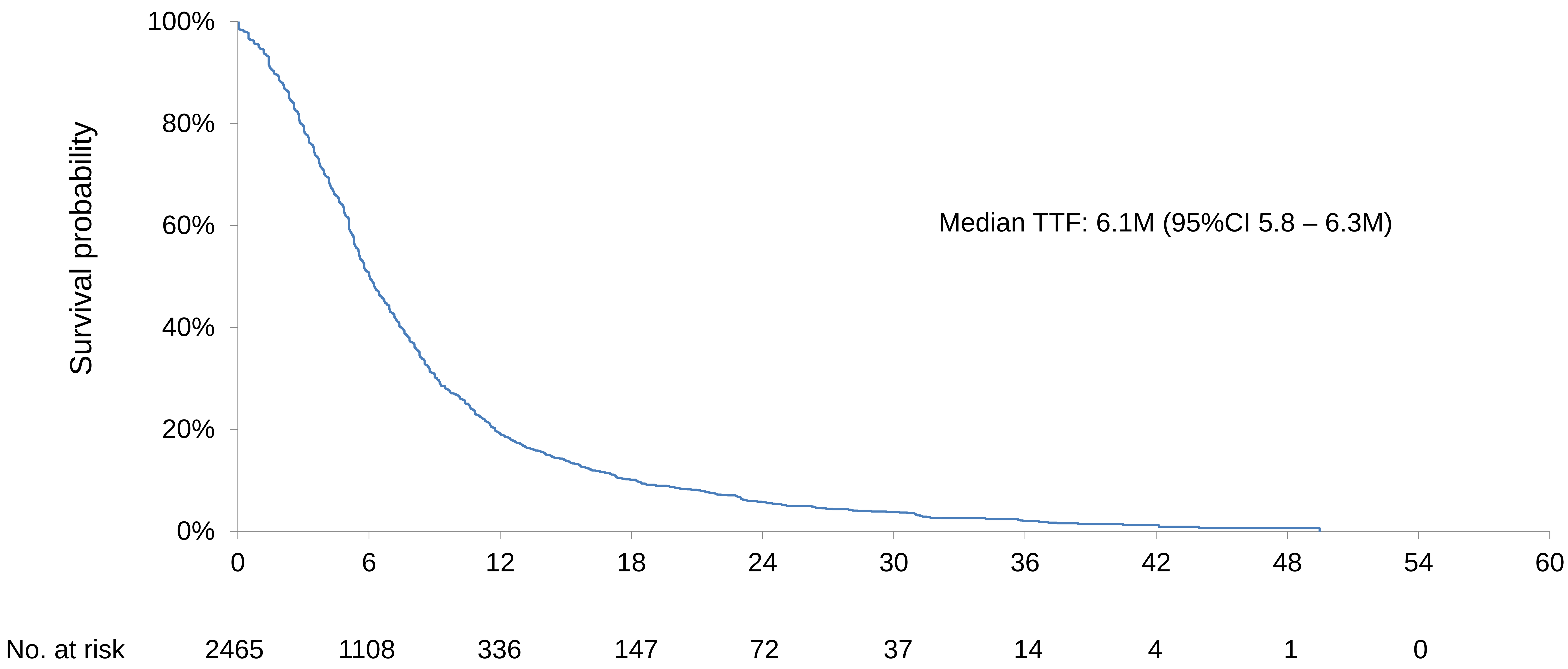

Supplementary figure 2a)

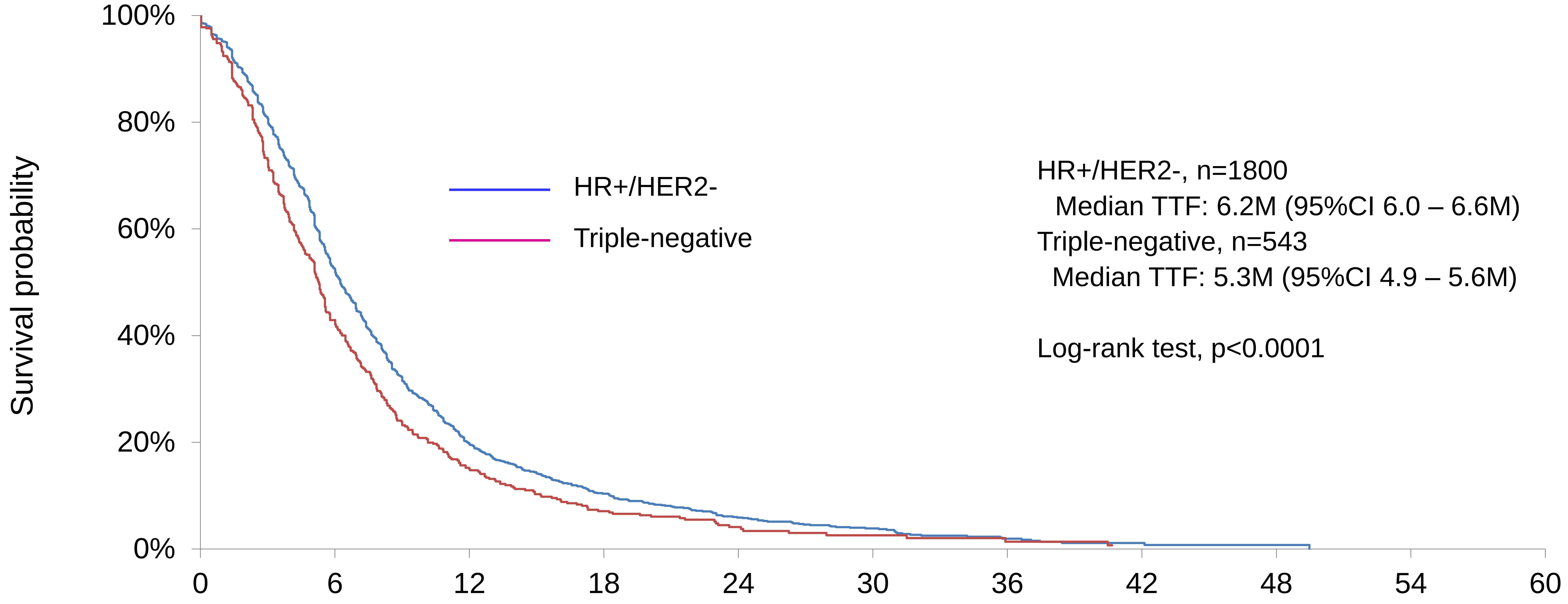

No. at risk

HR+/HER2-	1800	829	242	105	56	29	10	3	1	0
Triple-negative	543	209	65	28	11	5	2	0	0	0

Supplementary Figure 2b)
