# Supplementary material for: Factors affecting prognosis in patients treated with bevacizumab plus paclitaxel as first-line chemotherapy for HER2-negative metastatic breast cancer: an international pooled analysis of individual patient data from four prospective observational studies
Source: Breast Cancer. 2022 Sep 3;30(1):88–100. doi: 10.1007/s12282-022-01399-1 (PMC9813142; doi:10.1007/s12282-022-01399-1)
Supplement: Supplementary file 2 — Supplementary file2 (DOCX 43 KB) [file 12282_2022_1399_MOESM2_ESM.docx]

**Supplementary Table 1.** Overview of the observational studies used in this pooled analysis

| Study | JBCRG-C05 (B-SHARE) | ML21165 | ML21647 (AVAREGE) | ML22452 (AVANTI) |
| --- | --- | --- | --- | --- |
| Country | Japan | Germany | Hungary | Germany |
| No. of patients included in this analysis | 419 (155 institutions) | 785 (495 institutions) | 214 (34 institutions) | 1056 (282 institutions) |
| Period of enrollment | Nov. 2011 – Oct. 2014 | May 2007 –Sep. 2009 | Dec. 2007 – Mar. 2011 | Oct. 2009 – Feb. 2015 |
| Main inclusion criteria for the study | - HER2-negative LA/mBC - No history of second-line chemotherapy for LA/mBC | - mBC - aged > 18 years old - no prior chemotherapy for mBC | - Newly diagnosed mBC - Started first-line BV+PTX within the preceding 6 months | - Aged > 18 years old - previously untreated LA, recurrent or mBC |
| Median follow up time  (25 percentile, 75 percentile) | 19.9 months  (10.0, 31.6) | 15.8 months  (8.7, 28.8) | 8.8 months  (5.8, 13.1) | 7.4 months  (3.5, 12.3) |
| Treatment continued at the end of the observation period | 2(0.5%) | 47(6.0%) | 14(6.5%) | 363(34.4%) |
| Treatment discontinuation | 417 (99.5%) | 738(94.0%) | 200(93.5%) | 693(65.6%) |
| Reason for treatment discontinuation  Progression  Adverse event  Others | 217(52.0%)  130(31.2%)  70(16.8%) | 476(60.6%)  70(8.9%)  192(34.5%) | 113(52.8%)  33(15.4%)  54(25.2%) | 414(39.2%)  44(4.2%)  235(34.4%) |

BV, bevacizumab; HER2, human epidermal growth factor receptor-2; JBCRG, Japan Breast Cancer Research Group;LA, locally advanced; mBC, metastatic breast cancer; No., number; PTX, paclitaxel

**Supplementary Table 2. Patients’ characteristics**

|  |  | All patients | | B-SHARE | | ML21165 | | ML21647 (AVAREGE) | | ML22452 (AVANTI) | |
| --- | --- | --- | --- | --- | --- | --- | --- | --- | --- | --- | --- |
|  |  | *N* | (%) | *n* | (%) | *n* | (%) | *n* | (%) | *n* | (%) |
| No. of patients |  | 2474 | 100% | 419 | 100% | 785 | 100% | 214 | 100% | 1056 | 100% |
| Median age (range), years |  | 59.0 | (24–87) | 59.0 | (26–83) | 58.7 | (26–87) | 56.2 | (30–79) | 60.4 | (24-86) |
| ECOG PS |  |  |  |  |  |  |  |  |  |  |  |
|  | 0 | 1246 | 50.4% | 296 | 70.6% | 325 | 41.4% | 160 | 74.8% | 465 | 44.0% |
|  | 1 | 932 | 37.7% | 89 | 21.2% | 364 | 46.4% | 44 | 20.6% | 435 | 41.2% |
|  | > 2 | 201 | 8.1% | 34 | 8.2% | 73 | 9.2% | 7 | 3.3% | 88 | 8.4% |
|  | Missing | 95 | 3.8% | 0 | 0% | 24 | 3.0% | 11 | 5.1% | 68 | 6.4% |
| ER status of primary tumor |  |  |  |  |  |  |  |  |  |  |  |
|  | Negative | 667 | 27.0% | 104 | 39.6% | 201 | 25.6% | 113 | 52.8% | 249 | 23.6% |
|  | Positive | 1689 | 68.3% | 307 | 73.3% | 540 | 68.8% | 96 | 44.9% | 746 | 70.6% |
|  | Missing/Unknown | 118 | 4.7% | 8 | 1.9% | 44 | 5.6% | 5 | 2.3% | 61 | 5.8% |
| PgR status of primary tumor |  |  |  |  |  |  |  |  |  |  |  |
|  | Negative | 878 | 35.5% | 166 | 39.6% | 245 | 31.2% | 121 | 56.5% | 346 | 32.8% |
|  | Positive | 1463 | 59.1% | 243 | 58.0% | 486 | 61.9% | 88 | 41.1% | 646 | 61.2% |
|  | Unknown | 133 | 5.4% | 10 | 2.4% | 54 | 6.9% | 5 | 2.3% | 64 | 6.1% |
| HER2 status of primary tumor |  |  |  |  |  |  |  |  |  |  |  |
|  | Negative | 1994 | 80.6% | 399 | 95.2% | 541 | 68.9% | 206 | 96.3% | 848 | 80.3% |
|  | Positive | 161 | 6.5% | 2 | 0.5% | 80 | 10.2% | 5 | 2.3% | 74 | 7.0% |
|  | Missing/Unknown | 319 | 12.9% | 18 | 4.3% | 164 | 20.9% | 3 | 1.4% | 134 | 12.7% |
| Disease-free interval, months |  |  |  |  |  |  |  |  |  |  |  |
|  | < 24 (recurrence) | 412 | 16.7% | 101 | 24.1% | 126 | 16.1% | 54 | 25.2% | 131 | 12.4% |
|  | > 24 (recurrence)  or 0 (de novo) | 2026 | 81.9% | 318 | 75.9% | 627 | 79.9% | 157 | 73.4% | 924 | 87.5% |
|  | Missing | 36 | 1.4% | 0 | 0% | 32 | 4.1% | 3 | 1.4% | 1 | 0.1% |
| Metastatic site |  |  |  |  |  |  |  |  |  |  |  |
|  | Non-visceral | 631 | 25.5% | 113 | 27.0% | 181 | 23.1% | 74 | 34.6% | 263 | 24.9% |
|  | Visceral | 1843 | 74.5% | 306 | 73.0% | 604 | 76.9% | 140 | 65.4% | 793 | 75.1% |
| No. of metastatic organs |  |  |  |  |  |  |  |  |  |  |  |
|  | <3 | 2011 | 81.3% | 352 | 84.0% | 625 | 79.6% | 183 | 85.5% | 851 | 80.6% |
|  | >3 | 463 | 18.7% | 67 | 16.0% | 160 | 20.4% | 31 | 14.5% | 205 | 19.4% |
| Metastatic sites^a^ |  |  |  |  |  |  |  |  |  |  |  |
|  | Liver | 1029 | 41.6% | 158 | 37.7% | 352 | 44.8% | 65 | 30.4% | 454 | 43.0% |
|  | Lung | 939 | 38.0% | 154 | 36.8% | 290 | 36.9% | 96 | 44.9% | 399 | 37.8% |
|  | Central nervus system | 52 | 2.3% | 18 | 4.3% | 18 | 2.3% | 0 | 0% | 20 | 1.9% |
|  | Bone | 1353 | 54.7% | 220 | 52.5% | 446 | 56.8% | 101 | 47.2% | 607 | 57.5% |
|  | Bone only | 312 | 12.6% | 58 | 13.8% | 89 | 11.3% | 16 | 7.5% | 149 | 14.1% |
|  | Other | 957 | 38.7% | 193 | 46.1% | 283 | 36.1% | 91 | 42.5% | 390 | 36.9% |
| History of surgery for primary disease |  |  |  |  |  |  |  |  |  |  |  |
|  | No | 1179 | 47.7% | 7 | 1.7% | 60 | 7.6% | 214 | 100% | 898 | 85.0% |
|  | Yes | 1168 | 47.2% | 285 | 68.0% | 725 | 92.4% | 0 | 0% | 158 | 15.0% |
|  | Missing | 127 | 5.1% | 127 | 30.3% | 0 | 0% | 0 | 0% | 0 | 0% |
| History of adjuvant therapy |  |  |  |  |  |  |  |  |  |  |  |
|  | Chemotherapy | 1402 | 56.7% | 205 | 48.9% | 498 | 63.4% | 133 | 62.1% | 566 | 53.6% |
|  | Anthracycline (A) ^b^ | 1193 | 85.1% | 166 | 81.0% | 406 | 81.5% | 125 | 94.0% | 496 | 87.6% |
|  | Taxane (T) ^b^ | 686 | 48.9% | 151 | 73.7% | 189 | 38.0% | 50 | 38.0% | 296 | 52.3% |
|  | A and/or T ^b^ | 1239 | 88.4% | 186 | 90.7% | 419 | 84.1% | 127 | 95.5% | 507 | 89.6% |
|  | Endocrine therapy | 1280 | 51.7% | 191 | 45.6% | 494 | 63.4% | 132 | 61.7% | 463 | 43.8% |
|  | Radiotherapy | 1419 | 57.4% | 205 | 48.9% | 546 | 69.6% | 61 | 28.5% | 651 | 61.6% |

ECOG PS, Eastern Cooperative Oncology Group performance status; ER, estrogen receptor; HR, hormone receptor; PgR, progesterone receptor.
